# Dried strawberries as a high nutritional value fruit snack

**DOI:** 10.1007/s10068-018-0304-6

**Published:** 2018-01-24

**Authors:** Jolanta Kowalska, Hanna Kowalska, Agata Marzec, Tomasz Brzeziński, Kinga Samborska, Andrzej Lenart

**Affiliations:** 1 Department of Biotechnology, Microbiology and Food Evaluation, Division of Food Quality Evaluation, Faculty of Food Sciences, Warsaw University of Life Sciences - SGGW, 159c Nowoursynowska St, 02-776 Warsaw, Poland; 2 Department of Food Engineering and Process Management, Faculty of Food Sciences, Warsaw University of Life Sciences, 159c Nowoursynowska St, 02-776 Warsaw, Poland

**Keywords:** Strawberry, Osmotic dehydration, “Puffing”, Freeze-drying, Polyphenols, Vitamin C

## Abstract

The purpose of this study was to determine the possibility of using a chokeberry juice concentrate as a component of osmotic solution and convection-microwave-vacuum drying applying to obtain dried pro-health-promoting strawberries. The research material was *Honeoye* strawberries, which were dehydrated in sucrose and sucrose with chokeberry juice concentrate addition, and then subjected to microwave-convection-vacuum or freeze-drying. Analyses were conducted to determine the influence of the applied processes on vitamin C content, total polyphenols, antioxidant activity, and sensory properties in dried fruit. Study results confirmed the possibility of using a chokeberry juice concentrate as a component of the osmotic solution, especially with regard to polyphenolics content and antioxidant activity. In addition, convection-microwave-vacuum drying was shown to be a promising technology for the production of dried strawberries, with high pro-health potential and acceptable sensory qualities.

## Introduction

Seasonality is one of the key factors which determine the need for fruit processing primarily for juices, beverages and concentrates, but also for solids and frozen or dried products [1]. The popularity of strawberries is due to their sensory qualities as well as low caloric value, the content of easily digestible sugars, organic acids, minerals and also vitamins and ingredients with antioxidant properties [2, 3]. The health benefits of strawberries are determined by the abundance of biologically-active compounds that support the natural resistance of the organism, including ellagic acid (hexahydroxydiphenic acid), polyphenolics, mainly flavonoids, which help neutralize free radical damage, thereby reducing the risk of development of cardiovascular disease [4, 5].

Due to their short shelf life, strawberries are subjected to various technological processes, mainly freezing and also processing into jams or beverages. One of the most important methods is drying, which affords the possibility of extending the shelf life of strawberries, and also manufacturing new products. Regardless of the drying methods applied, high temperature applied in the drying process causes the loss of bioactive compounds and changes in sensory properties of fruit. Hence, it is essential to adjust a drying method to various raw materials—in terms of its type and parameters—to optimize properties of the dried fruit. In addition, pre-treatment processes such as osmotic dehydration with various solutions are used to ensure the desired nutritional and sensory properties of dried products [6]. The most commonly used osmotic substances are carbohydrates. Products with high sugar content are not advisable in fruit snacks production for nutritional reasons, therefore natural bioactive ingredients are used instead. The use of fruit juice concentrates and also fruit pomace extracts can allow manufacturing products enriched with bioactive components. Chokeberry is a fruit rich in antioxidants such as anthocyanins, flavanols or procyanidins, which also contains vitamins (C, B2, B6, E, P, PP). The use of chokeberry concentrate can enrich fruit with bioactive ingredients, partially lost in technological processes [7].

The aim of this study was to analyze the pro-health potential of chokeberry juice concentrate as an osmotic substance to enhance bioactive properties of dried strawberries produced with various drying techniques.

## Materials and methods

### Raw material

The study was conducted with strawberries of *Honeoye* variety at the stage of trade maturity (diameter of 25–30 mm), purchased directly from the manufacturer. The physiological state of the fruit indicated it was completely formed and grown, the most tasty, fully colored and firm. Sucrose was purchased at a local shop and chokeberry *Aronia melanocarpa* juice concentrate (CJC) of about 64°Brix [9870.6 mg of gallic acid/100 g dry matter; DPPH (2.2,-diphenyl-1-picrylhydrazyl) 50: 0.058] at RAUCH Polska Sp. z o.o. in Płońsk.

### Osmotic dehydration (OD)

OD of strawberries was carried out in solutions (sucrose and distilled water) at 50°Brix concentration. Solutions of sucrose and a mixture (Suc-CJC) of sucrose (Suc) and chokeberry juice concentrate (CJC) (diluted from 65 to 50°Brix) in a ratio of 1:1 (w:w) were used. The ratio of the weight of raw material to solution was constant and reached 2:1. The process was conducted in water bath with shaking (ELPAN-type 357) at temperature of 60 °C for 120 min, with continuous stirring at 1 Hz amplitude.

### Drying

Strawberries were dried without pre-treatment (control sample) or with OD and using different methods:

- the convection drying (120 min, 50 °C, air velocity 2 m/s) followed by microwave-vacuum drying (“puffing”) at: microwave power 400 W, pressure 35 hPa, time about 6 min, temperature 50–70 °C,
- the freeze-drying; first freezing in shock freezer at − 40 °C for 120 min followed by vacuum drying at 25 °C, 24 h, 100 Pa.

### Chemical analyses

Dry matter content of the samples was determined based on the standard PN-ISO-1026: 2000 [8].

The content of vitamin C in strawberries was determined spectrophotometrically at a wavelength of λ = 500 nm [9]. The principle of the method consists in the extraction of vitamin C with oxalic acid and its quantitative oxidation in the acidic medium to dehydroascorbic acid by the excess of 2,6-dichlorophenolindophenol. The results were calculated on the basis of the regression equation from the calibration curve and expressed as mg vitamin C per 100 g d.m. (dry matter).

The method with the Folin-Ciocalteu reagent [4] was used to determine the content of polyphenolic compounds in the samples of strawberries. Acetone extracts were prepared by adding 80% acetone to 2.0 g of fresh or 0.2 g of dried strawberries. The samples were homogenized and filtrated, and then distilled water and Folin-Ciocalteu reagent were added to the resultant extract. Afterwards, the samples were mixed and sodium carbonate was added. Thus prepared samples were kept for 60 min with no access of light. Absorption was measured with a Helios γ ThermoSpectronic spectrometer at wavelength of 750 nm against test zero. Standard curve was plotted for chlorogenic acid considering its various concentrations and absorption measurements. The total pholyphenols content was calculated in the samples based on the determined equation of the standard curve considering dilutions. Results were expressed in mg of chlorogenic acid per 100 g d.m. (dry matter).

The modified method proposed by Wu et al. [10] was used to determine the antioxidative potential of the samples, expressed by DPPH radical scavenging ability. Extracts were prepared like for determination of polyphenolics content. The basic DPPH extract was prepared by dissolving 1.2 mg of DPPH in 50 cm^3^ of methanol (99% of technical cleanliness), for which the zero test was prepared. The Helios γ ThermoSpectronic spectrometer was used for absorption measurement at a wavelength of 515 nm. The antioxidative activity of the analyzed extracts [A] was recalculated based on absorption measurement results of the exact and control test in Helios γ ThermoSpectronic spectrometer at the wavelength of 515 nm, against 99 v/v methanol.

Antioxidative activity against DPPH radicals was determined using the following equation:

$$ {\text{A}} = \frac{{{\text{A}}_{{{\text{c}} }} - {\text{A}}_{\text{a}} }}{{{\text{A}}_{\text{c}} }} \times 100\% $$

A_c_, control test absorbance, A_a_, applicable test absorbance. All assays were performed at least three times in parallel series, and each determination was conducted in five replications.

Sensory evaluation was carried out according to ISO 13299: 2016 [11] by out 15 panelist independent. The assessment was conducted on a 10-point scale with boundary conditions, where 1 meant the undesirable, least intense product, while 10 denoted the desirable product with the most desired overall sensory quality. The analyzed qualitative attributes were as follows: color—from red to dark red, with green spots visible, typical of the fruit; taste—sweet, with a slightly perceptible sour taste, juicy; flavor—slightly fruity, fresh fruit, characteristic for fresh strawberries; crispness—hardening—when pressed with a finger the product is not deformed, breaking/biting requires great force, overall sensory quality—acceptable—unacceptable.

The statistical analysis of results was conducted using a multi-way analysis of variance based on ANOVA (StatSoft-Statistica 12.0.). Two factors were investigated, the type of osmotic solution used for fruit pre-treatment and drying method, at a significance level of *p* = 0.05. Significant differences between means were determined with the Tukey’s Multiple Range Tests.

## Results and discussion

### The influence of convective with microwave-vacuum drying (“puffing”) and freeze-drying on dry matter content

The average dry matter content of fresh strawberries was 7.53% (Table 1). Such the low dry matter content means high water content that determines taste values, mainly juiciness of the fruit. The consumption of such fruit ensures good hydration of the body. Simultaneously, it is conducive to the development of microorganisms, hence storage of such fruit is limited [12]. Various methods of drying promote a significant loss of water in fruit. Osmotic pre-treatment and also the type of osmotic solution had a significant impact on dry matter content of dried strawberries (Table 1). Literature works described various extents of diffusion of osmotic substance particles as affected by their molecular weight [6]. Partial replacement of sucrose with the chokeberry juice concentrate resulted in a decrease in dry matter content (mainly sugar) in favor of monosaccharides naturally occurring in the fruit.

**Table 1:**
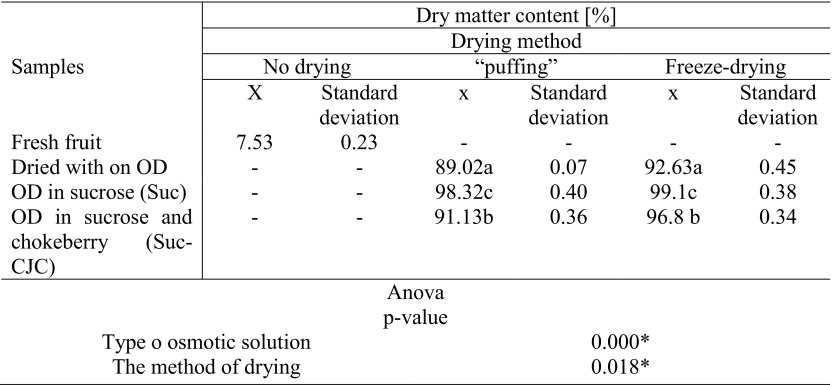
Dry mater content of osmo-dehydrated strawberries in different osmotic solutions and dried by freezing or convection with continues of microwave-vacuum method (“puffing”)

*Difference significant at *p* < 0.05

There was a significantly higher dry matter content in the freeze-dried samples compared to these subjected to convection followed by microwave-vacuum drying (“puffing”), which may be due to the principle of the method (Tab. 1). According to Sunjka et al. [1], Changrue et al. [13] and De Bruijn et al. [3], microwave energy is absorbed primarily by water contained in the material. The energy absorption by the wet material depends on its moisture distribution and causes selective heating of its interior parts, thus protecting low-moisture parts, e.g. material surface, from overheating [14]. Microwave heating causes volumetric heating and vapor is generated inside the product, developing internal pressure gradients that cause water flow from the interior to the surface of the material. In this way, food shrinkage is reduced [15].

The combined methods, including osmotic dehydration and the combination of microwave drying with convective drying, and the use of reduced pressure can shorten the time of drying and thus allow maintaining more components labile, e.g. vitamins, as well as the quality characteristics, such as no color changes [1, 16].

### The influence of convective with microwave-vacuum drying (“puffing”) and freeze-drying on vitamin C content

According Proteggente et al. [17] and Ramful et al. [20], strawberries can be classified as vitamin C-rich fruit. While the recommended daily intake for adults is 70 mg, vitamin C is non-toxic even in high doses, and hypervitaminosis of vitamin C has not been scientifically proven [21, 22].

Analyses showed about 490 mg of vitamin C in 100 g dry matter of fresh strawberries. These results are comparable to the values obtained by Wojdyło et al. [18], Giampieri et al. [23], and Gamboa-Santos et al. [19]. Because of the short shelf life of strawberries, resulting from the high water activity and associated microbial and enzymatic susceptibility, these fruit are often subjected to a variety of drying procedures. Due to high temperature effects of these procedures, the content of labile compounds is reduced, vitamin C in particular [12]. As demonstrated by Santos and Silva [24], Goula and Adamopoulos [25], and Nuñez-Mancilla et al. [26], vitamin C is the least resistant to external factors such as oxygen, light, temperature, pressure and exposure time. Therefore, many studies on food processing take vitamin C as an indicator of food quality.

**Fig. 1:**
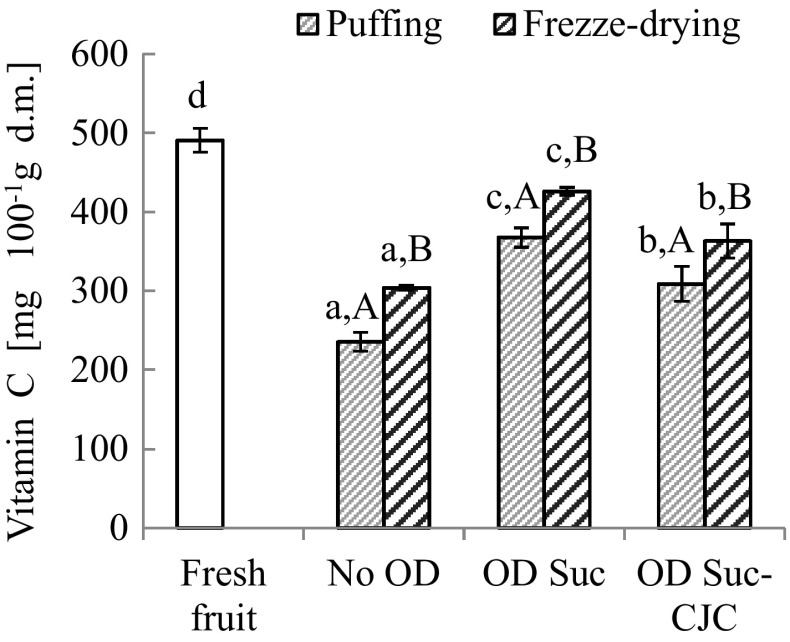
The influence of kind of osmotic solution and drying technics on the content of vitamin C in strawberry samples dried by “puffing” or freeze-drying way; no pre-treated (No OD), pre-osmotic dehydrated in sucrose (Suc) or in sucrose with chokeberry juice concentrate solution (Suc-CJC). The same letter, a, b, c, indicates a lack of statistically significant differences in kind of osmotic solution and A, B in drying technics

Due to the complexity and variety of factors which influence changes in vitamin C content in foods [26, 27], in the present study the changes in its content through initial osmotic dehydration and the application of two methods of fruit drying were compared. The tested dried strawberries had a significantly lower vitamin C content compared to fresh fruit (Fig. 1). The high concentration of the carbohydrate used for osmotic pre-treatment preserved vitamin C in the dried fruit (Fig. 1). A significantly higher vitamin C content (57–81% of freeze-dried and 19–40% of puffed) was determined in dried strawberries previously subjected to osmotic dehydration than in these with no pre-treatment.

Vitamin C content of strawberries was statistically significant affected by the type of osmotic solution applied. Strawberries dehydrated in a sucrose solution with the chokeberry juice concentrate contained by 17–19% more vitamin C then these dehydrated in sucrose with no additive. The relatively low content of vitamin C in chokeberry fruit (2.4 mg per 100 g fruit) has affected its low content in dried strawberries pre-dehydrated in the osmotic solution with the concentrate. In addition, some part of vitamin C was also lost during chokeberry fruit processing into a concentrate form.

Furthermore, this study demonstrates significantly higher vitamin C content in freeze-dried strawberries compared to these subjected to microwave-convective-vacuum drying (Fig. 1). According to Santos and Silva [24], citing Heng et al. [28] and Vial et al. [29], the protective effect on vitamin C may be attributed to the osmotic substance—sugar. As indicated by Santos and Silva [24], the mechanism of vitamin C degradation primarily depends on water content. Goula and Adamopoulos [25] indicated intensification of vitamin C losses along with a decrease in moisture content reaching up to 70%.

Apart from various factors affecting vitamin C degradation, as shown in numerous studies, its retention depends on the applied temperature, microwave power, pressure, type of pre-treatment (e.g. osmotic dehydration), and many others [26, 27]. Riva et al. [30] showed that the possibility of deactivating enzymes responsible for the oxidation and degradation of vitamin C under the influence of high temperatures (e.g. osmotic dehydration or microwave-convection drying), which can also prevent losses of this labile component. These studies have shown that freeze-drying at 25 °C allowed more vitamin C to be preserved, which may result from lower process temperatures and reduced oxygen availability at vacuum. Nevertheless, higher temperature and short time of the second technique of drying: 50 °C for 120 min during convection and 50–70 °C for about 6 min during microwave-vacuum drying, resulted in only 16–29% lower increase of vitamin C content in dried strawberries. Thus, the “puffing” method of fruit drying is highly beneficial considering vitamin C content of the dried fruit.

### The influence of convective with microwave-vacuum drying (“puffing”) and freeze-drying on polyphenolics content

The high antioxidative capacity of strawberries is related to their polyphenolics [31, 32]. Both technological processes as well as storage can affect changes in their content, i.e. some of them may reduce it while others may cause polyphenolics content to increase. In a research of De Bruijn et al. [3], it was shown that the metabolic pathways of polyphenolics can be stimulated by stress, e.g. modified storage conditions, with limited oxygen availability and without light. High temperatures can induce losses of polyphenolics, but the presence of proteins and carbohydrates in the nutritional matrix may protect against the effects of peroxidase and polyphenol oxidase, thereby protecting the product from losses of antioxidative compounds [3].

**Fig. 2:**
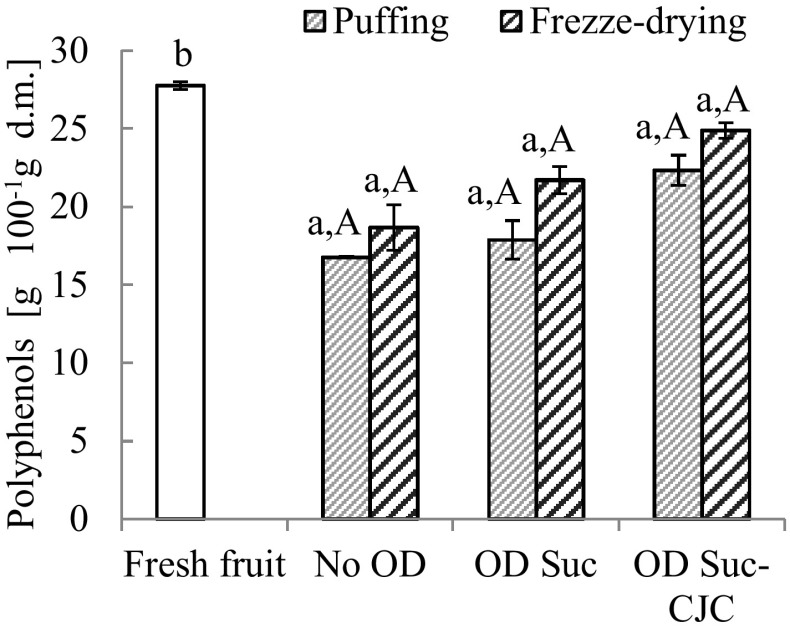
The influence of kind of osmotic solution and drying technics on the content of polyphenols in strawberry samples: dried by “puffing” or freeze-drying way; no pre-treated (No OD), pre-osmotic dehydrated in sucrose (Suc) or in sucrose with chokeberry juice concentrate solution (Suc-CJC). The same letter, a, b, c, indicates a lack of statistically significant differences in kind of osmotic solution and A, B in drying technics

The analyzed fresh *Honeoye* strawberries were characterized by a total polyphenols content of 27.7 g gallic acid in 100 g of the dry matter (Fig. 2). Strawberries are rich source of ellagitannins, anthocyanins, and procyanidins [33]. However, their contents differ among strawberry varieties, and additionally depend on crop conditions or storage conditions. The purpose of this study was to analyze changes in the content of selected bioactive components as a result of the applied osmotic dehydration and drying processes. As a result of osmotic pre-treatment and drying, the content of polyphenolics in strawberries reduced significantly, i.e. by 12–66% (Fig. 2). However, there was no statistically significant difference in the polyphenolics content, regardless of osmotic pre-treatment and drying technique. A tendency was observed for the greatest loss of polyphenolics in the no osmotically-dehydrated and only dried samples (16.8–18.7 g/100 g d.m.). The highest content of polyphenolics was determined in the dried strawberries osmotically pre-treated in sucrose solution with the addition of chokeberry juice concentrate (22.3–24.9 g/100 g d.m.).

When referring to the needs of the consumer, the change in total polyphenols content is expressed in terms of product weight (fresh or dried strawberries). The presence of polyphenols in plant tissues is a response to the stress induced by various factors including a rise or fall in temperature. Due to the effects of external factors, polyphenolics can undergo various changes—oxidation, hydrolysis or condensation. The least resistant to environmental changes are anthocyanins, while the predominant in strawberry ellagitannins behave in a different way [3, 33]. These hypotheses are reflected in the results of this study. The pre-osmotic treatment with a combination of convective and vacuum-microwave drying (“puffing”) was shown to be effective in preserving the heat- and oxygen-sensitive components such as polyphenolics and ascorbic acid of dried strawberries. However, the freeze-dried samples were characterized by the slightly higher total polyphenols content, which could support the beneficial effect of low temperatures on the behavior of these compounds.

Already 10 or more minutes of osmotic treatments at 60–65 °C resulted in the inactivation of polyphenol oxidase and peroxidase enzymes, which significantly reduced the oxidation of polyphenolics [3]. However, both the effect of elevated temperature and oxygen availability affected the loss of the total polyphenolics, especially anti-cyanide. The results obtained in this study confirm the above conclusions. The drying process affected the total polyphenols content of the analyzed samples. The protective effect of sugars on polyphenolic compounds has been demonstrated. Total polyphenols content in the sucrose-dehydrated samples was higher compared to the untreated samples (Fig. 2). The higher content of polyphenolics was also determined in the samples dehydrated in the sucrose solution with added chokeberry juice concentrate. The content of polyphenolics in freeze-dried strawberries after osmotic dehydration in a solution of chokeberry was by only about 11% lower than in the raw material. It confirms the validity of using chokeberry juice as a component of the osmotic solution to enrich dried fruit.

### The influence of convective with microwave-vacuum drying (“puffing”) and freeze-drying on antioxidative activity

Antioxidative activity is mainly due to the reducing of polyphenolics properties [34]. Based on preliminary studies [6], stable DPPH radicals have been used for this analysis.

**Fig. 3:**
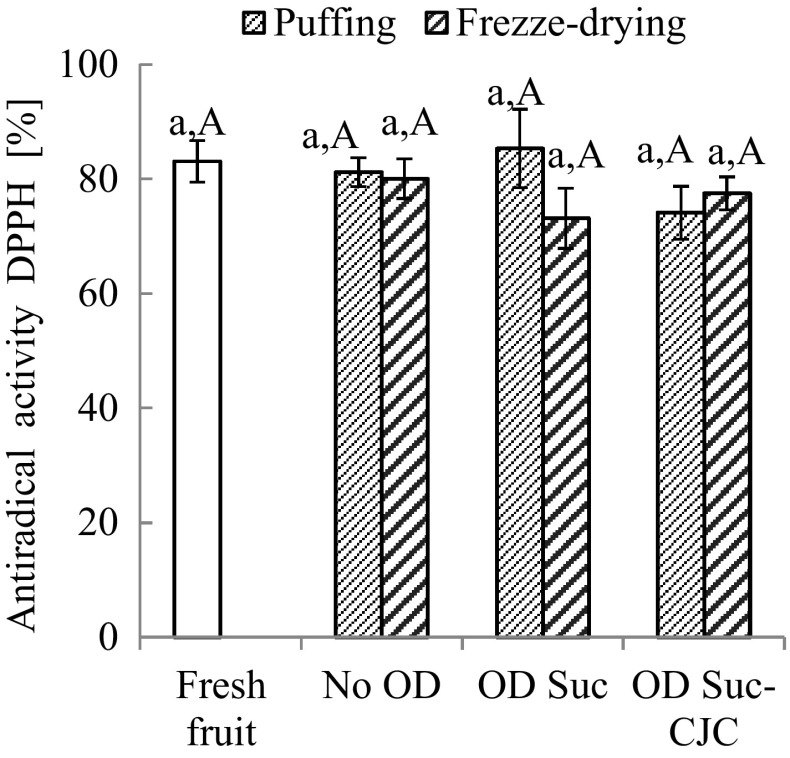
The influence of kind of osmotic solution and drying technics on the antiradical activity DPPH in strawberry samples dried by “puffing” or freeze-drying way; no pre-treated (No OD), pre-osmotic dehydrated in sucrose (Suc) or in sucrose with chokeberry juice concentrate solution (Suc-CJC). The same letter, a, b, c, indicates a lack of statistically significant differences in kind of osmotic solution and A, B in drying technics

There was no statistically significant difference in the antiradical activity, regardless of osmotic pre-treatment and drying technique. A tendency for the lowest value of this indicator was observed in the osmotically pre-treated samples dehydrated in a sucrose solution with chokeberry juice concentrate. According to Al-Musharfi et al. [34] citing Hossain et al. [35], there is a correlation, most commonly positive, between antioxidative activity and ascorbic acid content and polyphenolics content in fruit. This study demonstrated an opposite relationship between vitamin C content and total polyphenols content and antioxidative activity (Fig. 3). The statistical inference showed no correlation between vitamin C content and antioxidative activity against stable DPPH radicals (Table 2). The content of total polyphenolics was also not correlated with the antioxidative activity. Nevertheless, all analyzed samples were characterized by high antioxidative activity, demonstrating the ability to absorb and neutralize free radicals in the human body. Many authors showed a correlation between the antioxidative activity and polyphenolics content [35]. However, as demonstrated by Al-Musharfi et al. [33] despite its high value the antioxidative activity was not correlated with the polyphenolics content. Similar dependencies have been demonstrated in these studies.

**Table 2:** Correlations between chemical and sensory properties of dried strawberries

Factors	Dry matter	Vitamin C	Polyphenols	DPPH	Overal quality	Color*	Taste*	Flavor*
Dry matter	–	**−** 0.628*	**−** 0.655*	**−** 0.373	–	**−** 0.644*	**−** 0.551	**−** 0.959*
Vitamin C	**−** 0.628*	–	0.713*	0.037	0.497	0.499	0.000	0.498
Polyphenols	**−** 0.655*	0.713*	–	0.232	0.814*	0.186	0.287	0.674*
DPPH	**−** 0.373	0.037	0.232	–	0.191	0.340	**−** 0.016	0.465
Overal quality	**−** 0.874*	0.497	0.814*	0.191	–	0.352	0.705*	0.906*
Color*	**−** 0.644*	0.499	0.186	0.340	0.352	–	0.453	0.530
Taste*	**−** 0.551	0.000	0.287	**−** 0.016	0.705*	0.453	–	0.607*
Flavor*	**−** 0.959*	0.498	0.674*	0.465	0.906*	0.530	0.607*	–

The bold and * means the value of different significant at *p* < 0.05

### The influence of convective with microwave-vacuum drying (“puffing”) and freeze-drying on sensory properties

**Fig. 4:**
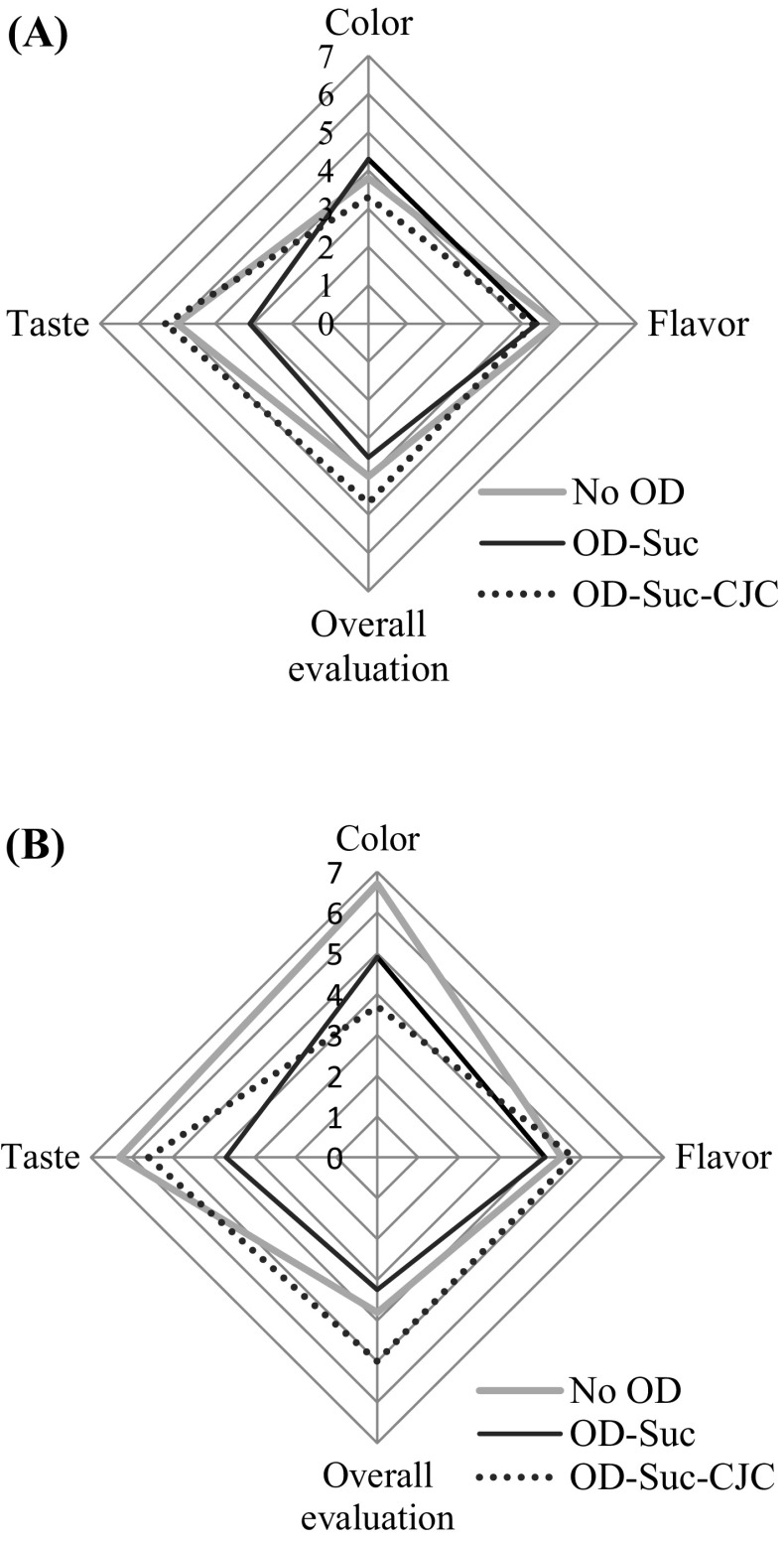
The influence of kind of osmotic solution and drying technics on sensory properties of strawberry samples dried by: (**A**) “puffing” (Puf) and (**B**) freeze-drying (FD) way; no pre-treated (No OD), pre-osmotic dehydrated in sucrose (Suc) or in sucrose with chokeberry juice concentrate solution (Suc-CJC)

Antioxidative properties that are important to the human/consumer body are also important for their preferences and acceptance of the product. Therefore, the sensory evaluation of was performed for the obtained samples in terms of their color, taste, flavor and overall quality (overall desirability). Sensory panel members indicated dried strawberries as highly desirable, with the high qualities tested (Fig. 4). Strawberries dried using convection-microwave-vacuum were evaluated higher than these subjected to freeze-drying treatment. Values of all the sensory attributers differed significantly as affected by pre-treatment, drying methods and osmotic substances. However, the scores were in fairly narrow ranges. In terms of taste, the highest scores (5.0–6.3) were given to the samples without pre-osmotic treatment and dried with both methods. Similar values (5.3–5.6) were obtained for the strawberries pre-osmotically dehydrated in the mixture of sucrose and chokeberry juice concentrate (Suc-CJC) solution, also depending on the drying method. The samples that were first osmo-dehydrated in the sucrose solution (Suc) and then dried respectively by “puffing” (3.1) and freeze-drying (3.7) were the least acceptable. Probably it was due to too much perceptible sweetness (Fig. 4). The color of freeze-dried strawberries was evaluated higher and in a much wider range of 3.7–6.7 than in these dried by “puffing” (3.8–4.3). Flavor of all dried fruits was scored high and at a very similar level (4.1–4.9). Depending on the drying method, some of the attributes were scored higher and others lower, although the highest desirability (overall sensory quality) was shown for the samples pre-dehydrated in the sucrose solution with chokeberry juice concentrate (Suc-CJC). It suggests that the products obtained in this way could be accepted by consumers.

Gamboa-Santos et al. [19] performed sensory evaluation of dried and rehydrated strawberries in different solutions and showed higher flavor and texture values for the freeze-dried fruit compared to the convection-microwave-vacuum dried samples. They explained these observations by higher porosity of freeze-dried strawberries and a more compact structure of fruit dried by “puffing”. However, considering the other sensory attributes analyzed, fruit obtained via both drying methods had similar notes.

Differences between properties of convection-microwave-vacuum and freeze-dried strawberries were not significant, which suggests that the products obtained by both methods could be accepted by consumers.

### A complex evaluation of the properties of dried strawberries

**Fig. 5:**
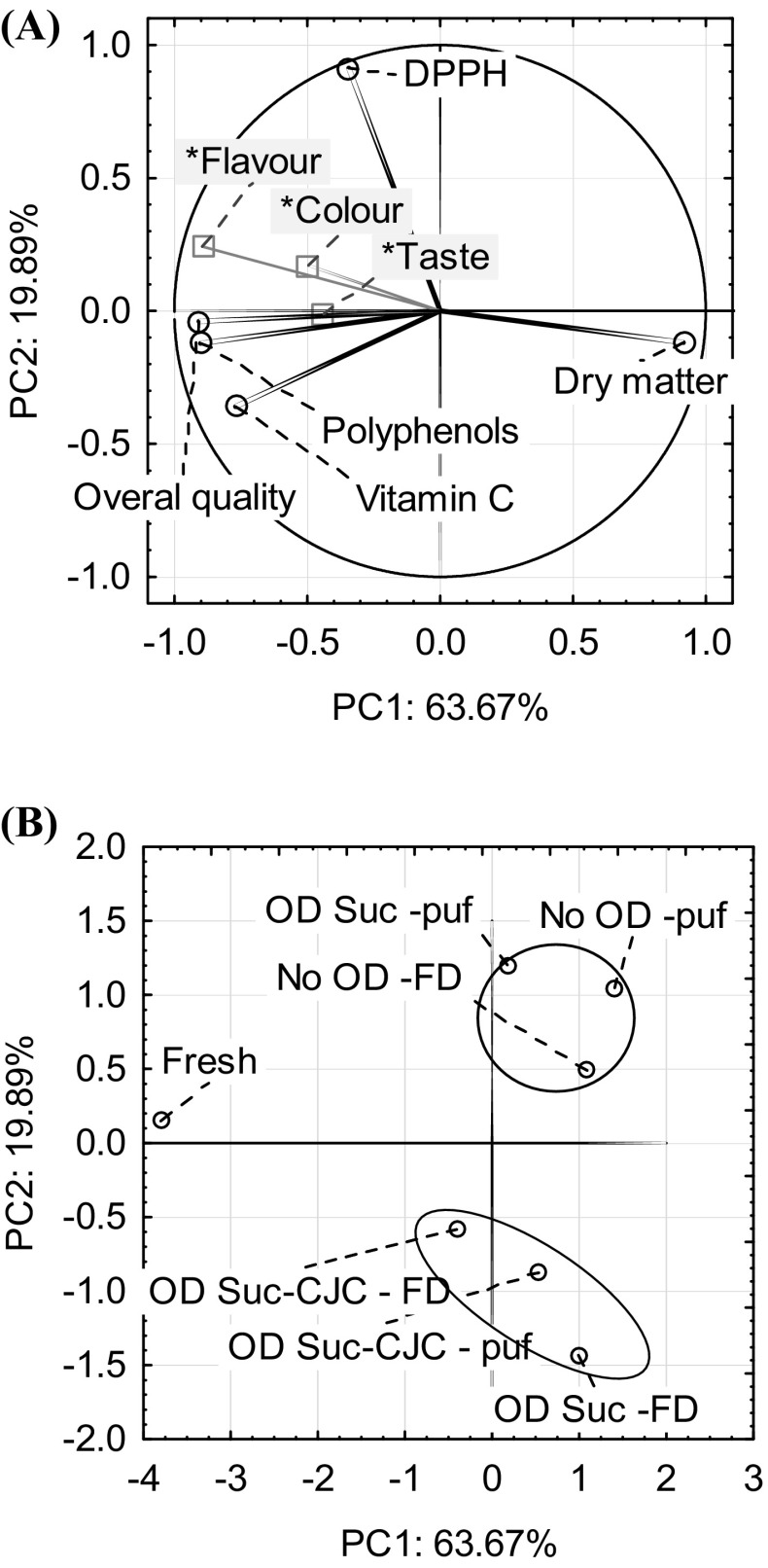
PCA analysis: (**A**) d*i*agram PCA, (**B**) similarities and differences

To determine similarities and differences between the analyzed types of dried strawberry samples in the aspect of the evaluated chemical and sensory properties, the main components were analyzed taking into account the mean values of the results obtained for each of the tested indicator [Fig. 5(A)].

The number of tested variables was reduced to two main PC1 and PC2 components, which explained 96.12% of the variation in pro-health and sensory properties of dried strawberries. A significant positive correlation was found between total polyphenols content of dried strawberries (snack) with other bio-component (vitamin C) and also sensory descriptors such as flavor, color and overall quality [Fig. 5(A), Table 2]. In contrast, a significant negative correlation was observed between dry matter content and some factors as vitamin C content, polyphenols content, and also overall quality, color and flavor. The negative correlation between vitamin C and dry matter contents is likely to result from vitamin C leaching during initial osmotic treatment and also from drying treatments [18].

The use of pre-osmotic dehydration with different types of osmotic solution allowed dividing the obtained data as in Fig. 5(B). The use of osmotic dehydration as a pre-treatment method, the type of osmotic solution, and the drying method applied had a significant impact on the division of results achieved into separate groups. Basically, the dried strawberries were essentially different in composition and sensory properties from fresh fruit. On the other hand, two other groups were distinguished mainly by the use of another osmotic substance for the initial dehydration of strawberries.

Strawberries are beneficial dietary sources of bioactive compounds, including polyphenolics as well as vitamin C. Due to the short availability of fresh fruit, it is reasonable to process strawberries to be available throughout the year.

Dried strawberries can be a good alternative to snacks satisfying not only the basic nutritional needs but also providing bio-components valuable for the human body. Sensory characteristics are also important because of product acceptance. Dried strawberries enriched with antioxidants derived from concentrated chokeberry juice with acceptable sensory properties may be a snack as well as an additive to other products. Microwave-convection-vacuum drying (“puffing”) is an alternative to freeze-drying.

Determinations of contents of polyphenolics and vitamin C in dried materials subjected to preliminary dehydration/enrichment and then drying may be useful indicators during development of technologies for the production of high-quality bio-snacks acceptable by consumers.

## References

[1] Sunjka PS, Rennie TJ, Beaudry C, Raghavan GSV (2004). Microwave-convective and microwave-vacuum drying of cranberries: a comparative study. Dry Technol.

[2] de Souza VR, Pereira PAP, da Silva TLT, de Oliveira Lima LC, Pio R, Queiroz F (2014). Determination of the bioactive compounds, antioxidant activity and chemical composition of Brazilian blackberry, red raspberry, strawberry, blueberry and sweet cherry fruits. Food Chem.

[3] De Bruijn J, Rivas F, Rodriguez Y, Loyola C, Flores A, Melin P, Borquez R (2015). Effect of vacuum microwave drying on the quality and storage stability of strawberries. J Food Process Preserv.

[4] Forbes-Hernandez TY, Gasparrini M, Afrin S, Bompadre S, Mezzetti B, Quiles JL, Giampieri F, Battino M (2016). The healthy effects of strawberry polyphenols: which strategy behind antioxidant capacity?. Crit Rev Food Sci Nutr Suppl.

[5] Giampieri F, Forbes-Hernandez TY, Gasparrini M, Afrin S, Cianciosi D, Reboredo-Rodriguez P, Varela-Lopez A, Quiles JL, Mezzetti B, Battino M (2017). The healthy effects of strawberry bioactive compounds on molecular pathways related to chronic diseases. Ann NY Acad Sci.

[6] Kowalska H, Marzec A, Kowalska J, Ciurzyńska A, Czajkowska K, Cichowska J, Rybak K, Lenart A (2017). Osmotic dehydration of *Honeoye* strawberries in solutions enriched with natural bioactive molecules. LWT Food Sci Technol.

[7] Buran TJ, Sandhu AK, Li Z, Rock CR, Yang WW, Gu L (2014). Adsorption/desorption characteristics and separation of anthocyanins and polyphenols from blueberries using microporous adsorbent resins. J Food Eng.

[8] PN-ISO1026:2000 Fruit and vegetable products—determination of dry matter content by drying under reduced pressure and of water content by azeotropic distillation

[9] PN-A-04019:1998—Produkty spożywcze—Oznaczanie zawartości witaminy C/Food products—determination of of vitamin C content (in Polish)

[10] Wu C, Huang M, Lin Y, Ju H, Ching H (2007). Antioxidant properties of Cortex fraxini and its simple coumarins. Food Chem.

[11] ISO PN-EN ISO 13299:2016 Sensory analysis—Methodology—General guidance for establishing a sensory profile

[12] Feguš U, Žigon U, Petermann M, Knez Ž (2015). Effect of drying parameters on physiochemical and sensory properties of fruit powders processed by PGSS-, vacuum and spray-drying. Acta Chim Slov.

[13] Changrue V, Raghavan GSV, Gariépy Y, Orsat V (2007). Microwave vacuum dryer setup and preliminary drying studies on strawberries. J Microw Power Electromagn Energy.

[14] Chandrasekaran S, Ramanathan S, Basak T (2013). Microwave food processing—a review. Food Res Int.

[15] Zhang M, Tang J, Mujumdar AS, Wang S (2006). Trends in microwave-related drying of fruits and vegetables. Trends Food Sci Technol.

[16] De Bruijn J, Borquez R (2014). Quality retention in strawberries dried by emerging dehydration method. Food Res Int.

[17] Proteggente AR, Pannala AS, Paganga G, van Buren L, Wagner E, Wiseman S, van de Put F, Dacombe C, Rice-Evans CA (2002). The antioxidant activity of regularly consumed fruit and vegetables reflects their phenolic and vitamin C composition. Free Radic Res.

[18] Wojdyło A, Figiel A, Oszmiański J (2009). Effect of drying methods with the application of vacuum microwaves on the bioactive compounds, color, and antioxidant activity of strawberry fruits. J Agric Food Chem.

[19] Gamboa-Santos J, Megías-Pérez R, Cristina Soria A, Olano A, Montilla A, Villamiel M (2014). Impact of processing conditions on the kinetic of vitamin C degradation and 2-furoylmethyl amino acid formation in dried strawberries. Food Chem.

[20] Ramful D, Tarnus E, Aruoma OI, Bourdon E, Bahroun T (2011). Polyphenols composition, vitamin C content and antioxidant capacity of mauritian citrus fruit pulps. Food Res Int.

[21] Kunachowicz H, Przygoda B, Nadolna I, Iwanow K. Tables of composition and nutritional value of food. Wyd. Lekarskie PZWL Press (2005) (in Polish)

[22] Płocharski W, Markowski J, Pytasz U, Rutkowski K (2013). Fruit, vegetables, juices, their caloric content and nutritional value against the demand for energy and nutrients Part 2. The nutritional and health value of permitted health claims. Sci Tech Mag Ferment Fruit Veg Ind.

[23] Giampieri F, Tulipani S, Alvarez-Suarez JM, Quiles JL, Mezzetti B, Battino M (2012). The strawberry: composition, nutritional quality, and impact on human health. Nutrition.

[24] Santos PHS, Silva MA (2008). Retention of vitamin C in drying processes of fruits and vegetables—a review. Dry Technol.

[25] Goula AM, Adamopoulos KG (2006). Retention of ascorbic acid during drying of tomato halves and tomato pulp. Dry Technol.

[26] Nuñez-Mancilla Y, Pérez-Won M, Uribe E, Vega-Gálvez A, Di Scala K (2013). Osmotic dehydration under high hydrostatic pressure: effects on antioxidant activity, total phenolics compounds, vitamin C and color of strawberry (*Fragaria vesca*). LWT Food Sci Technol.

[27] Barba FJ, Esteve MJ, Frigola A (2011). Physicochemical and nutritional characteristics of blueberry juice after high pressure processing. Food Res Int.

[28] Heng K, Guilbert S, Cuq JL (1990). Osmotic dehydration of papaya: influence of process variables on the product quality. Sci Des Aliments.

[29] Vial C, Guilbert S, Cuq JL (1991). Osmotic dehydration of kiwi fruits: influence of process variables on the color and ascorbic acid content. Sci Des Aliment.

[30] Riva M, Campolongo S, Leva AA, Maestrelli A, Torreggiani D (2005). Structure-property relationships in osmo-air-dehydrated apricots cubes. Food Res Int.

[31] Panico A, Garufi F, Nitto S, Di Mauro R, Longhitano R, Magri G, Catalfo A, Serrentino M, De Guidi G (2009). Antioxidant activity and phenolic content of strawberry genotypes from Fragaria x ananassa. Pharm Biol.

[32] Concha-Meyer AA, D’Ignoti V, Saez B, Diaz RI, Torres CA (2016). Effect of storage on the physico-chemical and antioxidant properties of strawberry and kiwi leathers. J Food Sci.

[33] Lipińska L, Klewicka E, Sojka M (2014). The structure, occurrence and biological activity of ellagitannins: a general review. Acta Sci Pol Technol Aliment.

[34] Al-Musharfi NK, Al-Wahaibi HS, Khan SA (2015). Comparison of ascorbic acid, total phenolic content and antioxidant activities of fresh juices of six fruits grown in Oman. J Food Process Technol.

[35] Hossain SJ, El-Sayed M, Aoshima H (2009). Antioxidative and anti α-amylase activities of four wild plants consumed by pastoral nomads. Egypt Orient Pharm Exp Med.

